# Temporomandibular disorders in migraine and tension-type headache patients: a systematic review with meta-analysis

**DOI:** 10.22514/jofph.2024.011

**Published:** 2024-06-12

**Authors:** Paolo Bizzarri, Daniele Manfredini, Michail Koutris, Marco Bartolini, Luca Buzzatti, Cecilia Bagnoli, Aldo Scafoglieri

**Affiliations:** Experimental Anatomy Research Group (EXAN), Vrije Universiteit Brussel (VUB), 1090 Brussels, Belgium; Department of Human Neuroscience, University of Rome La Sapienza, 00185 Rome, Italy; Department of Clinical Science and Translational Medicine, University of Rome “Tor Vergata”, 00133 Roma, Italy; Department of Biomedical Technologies, School of Dentistry, University of Siena, 53100 Siena, Italy; Department of Orofacial Pain and Dysfunction, Academic Centre for Dentistry Amsterdam (ACTA), University of Amsterdam and Vrije Universiteit Amsterdam, 1081 LA Amsterdam, Netherlands; Clinic of Neurology, Polytechnic University of Marche, 60126 Ancona, Italy; School of Allied Health, Anglia Ruskin University, CB1 1PT Cambridge, UK

**Keywords:** Primary headaches, Orofacial pain, Association, Temporomandibular joint, Masticatory muscles, Epidemiology

## Abstract

The simultaneous occurrence of primary headaches and temporomandibular disorders can pose a challenge in determining the best clinical management of patients. Therefore, we aimed to summarize evidence regarding the risk and prevalence of temporomandibular disorders (TMDs) in migraine and tension-type headaches (TTH) patients. Cross-sectional studies published in English comparing the presence of TMDs in adults with TTH or migraine to subjects without headaches were included, International Classification of Orofacial Pain, Diagnostic Criteria for Temporomandibular Disorders or Research Diagnostic Criteria for Temporomandibular Disorders, and large epidemiological studies (sensitive diagnostic criteria (SDC)). The methodological quality was assessed by Modified Newcastle-Ottawa Quality Assessment Scale. Odds ratio (OR) and random effects were calculated. 1405 articles were identified in PubMed, Embase and Central databases, and 13 cross-sectional studies were finally included. Overall Risk of TMDs was statistically significantly higher than control groups in both Migraine (SDC: 11 studies; OR: 3.79 (2.43, 5.90); *I*^2^ = 99%), with higher values in chronic migraine (OR: 24.27; (95% Confidence interval (CI): 5.84, 100.82); *I*^2^ = 0%) and TTH populations (SDC: 8 studies; OR: 4.45 (2.63, 7.53); *I*^2^ = 86%). Headache subjects presented a higher risk of muscular TMDs (5 studies; OR: 2.01 (1.62, 2.50); *I*^2^ = 0%), Combined TMDs (5 studies; OR: 2.74 (1.40, 5.36); *I*^2^ = 63%), or Painful TMDs (8 studies; OR: 5.31 (2.96, 9.54); *I*^2^ = 96%). Headache patients didn’t show the risk of arthrogenous TMDs (4 studies; OR: 0.96 (0.54, 1.71); *I*^2^ = 33%) or nonpainful TMDs (2 studies; OR: 1.10 (0.28, 4.26); *I*^2^ = 84%). The high heterogeneity in the results was reduced following subgroup analysis. Migraine and TTH appear to increase the risk of painful, myogenous or combined arthrogenous and myogenous TMDs.

## 1. Introduction

Headaches are described as the most disabling neurological condition worldwide. Tension-type headache (TTH) is the most prevalent form of headache (22%) and migraine is the most disabling one particularly among females aged 15–49 years [1]. TTH and migraine represent the vast majority of primary headaches, as defined in the International Classification of Headache Disorders (ICHD-3) [2]. Genetic, systemic, hormonal, environmental, psychological and physical factors appear to play a role in the clinical presentations and burden of these disorders. These factors influence peripheral and central mechanisms involved in head and face pain processing, in particular related to the trigeminocervical complex [3, 4, 5], facilitating segmental and general comorbidities. People with headaches often report sleep disorders, anxiety, depression and painful comorbidities, both in surrounding areas (face and neck pain) and widespread regions (back pain and fibromyalgia) [6, 7, 8]. Moreover, the risk of suffering from headaches has been described to increase from twofold to threefold in adult women compared to men [9], supporting the role of hormonal factors (mainly related to estrogens and progesterone) [10]. Female sex, sleep disorders, psychological and painful comorbidities are also risk factors for temporomandibular disorders [11, 12, 13, 14].

Temporomandibular disorders (TMD) are defined as a group of musculoskeletal and neuromuscular conditions that involve the temporomandibular joints (TMJs), the masticatory muscles and all associated tissues. As described in the Axis I of the Diagnostic Criteria for Temporomandibular Disorders (DC/TMD) [15], the main TMD categories are temporomandibular joint and masticatory muscle disorders, the prevalence of which ranges between 5% and 12% [16]. The distinction between these categories is based mainly on painful symptoms, biomechanical signs and structural alterations. Nociceptive signals from such peripheral structures, mainly innervated by the mandibular branch of the trigeminal nerve, can sensitize the trigeminocervical complex and modulate head pain [5]. The International Classification for Headache Disorders includes the condition. Headache attributed to temporomandibular disorder (TMD), a secondary headache caused by a disorder involving structures in the temporomandibular region (ICHD).

Even though isolated facial pain has been described as rare in primary headache populations [17], TMDs appear to be more prevalent in headache populations compared to non-headache subjects. Likewise, headache has been described as one of the most common comorbidities in subjects with TMD [18]. This overlap, often overlooked in clinical practice, can be challenging and critical in determining the best management of patients as well as in achieving a proper diagnosis early in the assessment phase. The presence of painful TMDs in subjects with primary headaches has been associated with higher headache frequency in adolescents [19]. However, the available literature lacks strong evidence that clarifies the relationship between primary headaches and TMDs. Most articles do not apply reference standards, such as DC/TMD [15], ICHD-3 [2] and ICOP [20], for categorizing patients. Moreover, reported data often refer exclusively to signs and symptoms, and lack non-headache control groups, inadvertently reducing the external validity of these findings.

Yakkaphan *et al*. [21] recently published a systematic review on the reciprocal association between TMDs and headaches. This review included studies without reference standard diagnostic criteria, and lacks the analysis of subgroups of primary headaches (*e.g.*, chronic forms). No systematic review has been published on this subject related to reference diagnostic criteria and large epidemiological studies. Therefore, we aimed to compare the risk of specific TMDs diagnoses in migraine and TTH patients and non-headache adult populations.

## 2. Materials and methods

The guidelines of the Meta-analysis of Observational Studies in Epidemiology (MOOSE) group [22] and the Preferred Reporting Items for Systematic Reviews and Meta-Analyses (PRISMA) reporting guidelines [23] were followed for this review. The protocol of this systematic review was registered in the PROSPERO database (CRD42020167535).

### 2.1 Data sources

We aimed to include cross-sectional studies published in English comparing the presence of specific TMD diagnoses in adults with primary headaches to adults without headaches. Preliminary electronic searches retrieved no studies with Trigeminal autonomic cephalalgias (TACs) or other primary headache disorders except for Migraine and Tension-Type Headaches (TTH). Therefore, this study and the search strategy focused on the latter two. A first electronic search was performed in PubMed, Embase and Central databases, on 19 September 2020. A second search was performed on 09 May 2022. The full search strategy is available in **Supplementary material**.

### 2.2 Study selection

No time restriction was applied to search strategies. The reference lists of the included studies were searched to retrieve supplemental primary articles of interest, using Rayyan website (https://www.rayyan.ai/) [24].

To be included, studies were required to be cross-sectional studies reporting data on the prevalence of diagnosis of TMD in a group of subjects with a specific diagnosis of migraine, TTH and a control group of non-headache subjects.

Studies and data regarding Chronic Daily Headache or headache symptoms without specific diagnoses were excluded for better adherence to ICHD-3 [2]. However, we didn’t restrict headache diagnoses specifically to ICHD criteria with the aim of including population-based studies. If a study reported data regarding both migraine or TTH, and other headaches, only data respecting our inclusion criteria were extracted.

Case reports, studies based on adolescent and paediatric populations, and studies regarding Infrequent Episodic TTH (IETTH) were also excluded to improve the external validity of our findings.

Due to the high variability of TMD classifications, and in order to simplify data analysis, we accepted diagnostic criteria for TMD as described in the single studies, if they respected our inclusion criteria. However, the term “facial pain” was not considered, given its possible overlap with clinical presentation of odontogenic pain, trigeminal neuralgia, migraine and TTH patients [25].

In preparing and summarizing our findings, we aimed to adhere to ICOP terminology and diagnostic disorders [20].

### 2.3 Study inclusion and data extraction

Two independent reviewers (PB, CB) screened all articles by title, abstract and full texts using Rayyan website [24]. Disagreements were resolved by consensus or by consulting a third investigator (LB). For each study, two reviewers independently extracted data regarding study design, setting, age, gender, diagnostic criteria and modalities, the total number of subjects with specific headaches and control groups, the total number of subjects with and without TMD diagnosis, signs and symptoms. In need of clarifications, especially regarding the inclusion of data in subgroup analyses, corresponding authors of retrieved studies were contacted *via* email. Two reviewers (PB, CB) assessed study quality using the Modified-Newcastle Ottawa Scale (M-NOS) for cross-sectional studies [26]. The tool assesses three domains: selection (four items), comparability (two items) and exposure (three items). A total of nine points are awarded, with higher sum scores reflecting superior study quality.

### 2.4 Data synthesis

The results of individual studies comparing the presence of TMD in subjects with and without primary headaches were pooled using the Mantel-Haenszel method with a random-effects model. Odds Ratio (OR) was chosen as the effect measure and corresponding 95% confidence intervals (CI) were calculated. Forest plots were created for graphical representation of the results. Heterogeneity was assessed by inspection of the CI from the forest plots, Chi-squared tests for heterogeneity and *I*^2^ statistics (<50% representing low heterogeneity, between 50% and 75% representing moderate heterogeneity, and greater than 75% representing high heterogeneity) [27]. Subgroup analysis was performed using different categories of primary headaches (tension-type headaches and migraine) and further stratification by type of TMD (joint, muscular or combined). In order to verify whether subgroup results were genuinely different, tests for subgroup differences were computed (*p*-value, *I*^2^). All analyses were performed in Review Manager 5.3 (RevMan—Copenhagen: The Nordic Cochrane Center, The Cochrane Collaboration, 2014, Denmark).

## 3. Results

The first database search identified 1405 results. After the removal of duplicates, and title and abstract screening, 92 full texts were examined. The second search was performed on 09 May 2022, retrieving 93 additional articles. No additional study was eligible. As described in the PRISMA flow chart (Fig. 1), 13 [28, 29, 30, 31, 32, 33, 34, 35, 36, 37, 38, 39, 40] cross-sectional studies met our inclusion criteria and were included in our qualitative synthesis. The main causes of exclusion were related to a non-specific diagnosis of headache, or the lack of subjects without headaches in the control group. Analysis of publication bias is reported in Fig. 2. The exclusion of the outlier [32] did not produce changes in the heterogeneity of results.

**Fig. 1.** PRISMA search-flow diagram.

**Fig. 2.** Funnel plot for publication bias of all studies included. OR: Odds Ratio; SE: Standard Error.

The following information was extracted from each publication selected for inclusion: Setting, Design, Inclusion Criteria, Headache (HA) and TMD diagnostic criteria, HA and TMD prevalence, age, gender distribution. Prevalence of temporomandibular disorders in patients with primary headaches and controls was computed for each study.

### 3.1 Characteristics of the studies

Headache diagnoses were made following ICHD criteria in 8 studies, whereas Research Diagnostic Criteria (RDC/TMD) or Diagnostic Criteria for Temporomandibular Disorders (DC/TMD) were used in 7 studies. ICOP classification was not performed in the included studies. 9 studies applied questionnaires for headache or TMD diagnoses. Thus, the terminology was not consistent throughout the articles. Five articles applied gold standard diagnostic classifications for both primary headaches and TMDs, thus results from these studies will be presented separately. To simplify data analysis, however, we accepted diagnostic criteria as described in single studies, if they respected our inclusion criteria. Full data descriptions are reported in Tables 1,2,3.

**Table 1.** Overview of included studies that applied reference classifications for primary headache and TMD diagnoses.

Author and Year	Country	Setting	Sample Size	Diagnostic Criteria for HA	Headaches Included	Diagnostic Criteria for Temporomandibular disorders	TMD Included
Franco 2010	Brazil	TMD and Orofacial Pain Clinic	n. HA: 161 n. Control: 37	ICHD-II Based Questionnaire	• Migraine • TTH	RDC/TMD	• Myofascial Pain • Arthralgia • Combined
Goncalves 2011	Brazil	University—based specialty clinic.	n. HA: 235 n. Control: 65	Questionnaire based on ICHD-II	• Migraine • ETTH	RDC/TMD—Questionnaire and Physical Examination	• jTMD • mTMD Combined TMD
Goncalves 2013	Brazil	University—based headache outpatient clinic (tertiary care)	n. HA: 61 n. Control: 30	ICHD-II—Neurologist	• Migraine • Chronic Migraine	RDC/TMD Axis I—Physiotherapists	• Group I • Group I + II • Group I + III Group I + II + III
Van der Meer 2017	Netherlands	Clinic for TMD and orofacial pain of the academic centre for dentistry, Amsterdam	n. HA: 137 n. Control: 66	ICHD-3—Headache Screening Questionnaire. Migraine and TTH	• Migraine • Probable Migraine • TTH • Probable TTH • HA attributed to TMD	RDC/TMD	• Painful TMD • Function related TMD
Wagner 2019	Brazil	Orofacial Pain Clinic	n. HA: 90 n. Control: 72	Neurologist—Questionnaire on ICHD-3 beta	Frequent TTH	RDC/TMD	• Painful TMD • Non-Painful TMD

*HA: Headache; ICHD: International Classification of Headache Disorders; TMD: Temporomandibular Disorders; jTMD: Joint TMD; mTMD: Myogenous TMD; TTH: Tension-Type Headache; ETTH: Episodic TTH; RDC/TMD: Research Diagnostic Criteria for Temporomandibular Disorders.*

**Table 2.** Overview of included studies that applied sensitive diagnostic criteria for primary headache and TMD diagnoses.

Author and Year	Country	Setting	Sample Size	Diagnostic Criteria for HA	Headache Included	Diagnostic Criteria for Temporomandibular disorders	TMD Included
Ashraf 2019	Finland	National survey	n. HA: 498 n. Control: 5378	Previous Diagnosis	• Migraine	Clinical Examination based on RDC/TMD	• jTMD • mTMD • Combined TMD
Benoliel 2010	Israel	Medical and dental students	n. HA: 299 n. Control: 60	Questionnaire based on HIS (ICHD-2)	• Migraine • FETTH • IETTH • CTTH	Questionnaire based on AAOP and RCD/TMD Criteria	• jTMD • mTMD • Combined TMD
Fenton 2016	USA	Musculoskeletal Disorder Cohort (MSD) cohort for Veterans	n. HA: 167,597 n. Controls: 3,960,391	Previous Diagnosis	• Migraine • Tension Headache	TMD was defined as ICD codes 524.29, 524.6, 524.60, 524.61, 524.62, 524.63, 524.64 and 524.69	• TMD
Florencio 2017	Brazil	Tertiary university based hospital	n. HA: 52 n. Control: 32	ICHD-III—Examination (Neurologist)	• Episodic Migraine • Chronic Migraine	Fonseca Anamnestic Index	• Mild TMD • Severe TMD
Goncalves 2010	Brazil	Urban Population	n. HA: 472 n. Control: 676	Questionnaire based on ICHD-II	• Migraine • ETTH	Questionnaire based on AAOP	• TMD
Plesh 2012	USA	National Survey—US. National Health Interview Survey (NHIS)	n. HA: 29,712 n. Control: 160,255	Questionnaire	• Migraine/Severe Headache	Questionnaire	TMJMDs Pain—Temporomandi-bular joint and muscle disorders pain
Song 2018	South Korea	Dataset of national survey	n. HA: 3940 n. Control: 17,575	Previous History in Survey	• Migraine	Signs and Symptoms	TMD
Schur 2007	USA	University of Washington Twin Registry (UWTR)—community-based registry	n. HA: 315 n. Control: 3622	Questionnaire on Previous Diagnosis	• Chronic Tension Headache	Questionnaire on Previous Diagnosis	• TMJ Syndrome

*HA: Headache; ICHD: International Classification of Headache Disorders; TMJ: Temporomandibular Joint; TMD: Temporomandibular Disorders; jTMD: Joint TMD; mTMD: Myogenous TMD; TTH: Tension-Type Headache; FETTH: Frequent Episodic TTH; IETTH: Infrequent Episodic TTH; CTTH: Chronic Tension-Type Headache; RDC/TMD: Research Diagnostic Criteria for Temporomandibular Disorders; AAOP: American Academy of Orofacial Pain.*

**Table 3.** Methodological quality of the studies.

Cross-sectional studies								
Study	Selection				Comparability		Outcome	
	Represen-tativeness of the sample	Sample size	Nonres-pondents	Ascertainment of the exposure	The study controls for the most important confounding factor	The study controls for any additional confounding factor	Assess-ment of outcome	Statistical test
Ashraf, 2019	⋆			⋆	⋆	⋆	⋆⋆	⋆
Benoliel, 2010				⋆⋆			⋆	⋆
Fenton, 2018	⋆			⋆⋆	⋆		⋆⋆	⋆
Florencio, 2017	⋆	⋆		⋆⋆			⋆	⋆
Goncalves, 2009	⋆	⋆		⋆⋆			⋆	⋆
Goncalves, 2013				⋆⋆			⋆⋆	⋆
Jussila, 2018				⋆			⋆⋆	⋆
Van Der Meer, 2017	⋆	⋆	⋆		⋆	⋆	⋆	⋆
Plesh, 2012	⋆			⋆	⋆	⋆	⋆	⋆
Schur, 2007	⋆			⋆	⋆	⋆	⋆	⋆
Song, 2018	⋆			⋆	⋆	⋆	⋆	⋆
Steele, 1991	⋆			⋆			⋆⋆	⋆
Wagner, 2019				⋆⋆			⋆	⋆

*Stars are assigned for a study’s design characteristics. Studies that garner more stars are deemed to be of higher quality.*

### 3.2 Quality of the studies

The methodological quality scores ranged from 4 to 7 out of a maximum of 9 (Table 3).

### 3.3 TMD risk in headache populations

The following presentation of TMD prevalence does not discriminate between specific TMD clinical characteristics. Specific subgroup analyses are shown afterwards in this article.

#### 3.3.1 TMD in headache patients—reference classifications

Five studies [28, 29, 30, 31, 32], all of which were clinically based, used a reference classification for both primary headaches (ICHD-3 or ICHD-2) and TMDs (RDC/TMD). Overall risk of suffering from TMDs was significantly higher in headache subjects than in non-headaches control groups (Prevalence: 82.9% *vs.* 42.4% OR 8.11 (95% CI: 2.58, 25.48); *I*^2^ = 91%) (Fig. 3).

**Fig. 3.** Overall risk of TMD in headache patients (Reference Classifications). CI: confidence intervals.

TMDs are also more prevalent in migraine patients compared to control groups (4 studies; 82.4% *vs.* 52.3%; OR 4.78 (95% CI: 2.16, 10.58); *I*^2^ = 69%) (Fig. 4), so as in TTH subjects (4 studies; 82.96% *vs.* 43.51%; OR 7.41 (95% CI: 1.50, 36.57); *I*^2^ = 87%) (Fig. 5).

**Fig. 4.** Risk of TMD in migraine (Reference Classifications). CI: confidence intervals.

**Fig. 5.** Risk of TMD in TTH (Reference Classifications). CI: confidence intervals.

#### 3.3.2 TMD in headache patients following sensitive diagnostic criteria

##### 3.3.2.1 Overall TMD risk in Migraine—sensitive diagnostic criteria

Irrespective of diagnostic criteria, 11 studies analysed the prevalence of TMD diagnosis in migraineurs compared to non-headache subjects. Studies were heterogeneous regarding setting, data collection and diagnostic criteria and modalities. Most studies didn’t differentiate migraine types. One study [36] distinguished between episodic and chronic migraine, one study [31] between migraine and probable migraine, and one study [30] between migraine and chronic migraine.

The pooled prevalence of TMD was 6.7% in migraineurs, whereas the prevalence of TMD in the non-headache population was 0.4%, with OR 3.79 ((95% CI: 2.43, 5.90); *I*^2^ = 99%).

One clinically based study [36] reported a prevalence of TMD of 77.4% in episodic migraine patients, compared to 53.1% in non-headache subjects, with OR 3.03 (95% CI: 1.02, 9.01). For chronic migraine, two clinically based studies [30, 36] observed a pooled prevalence of TMD in 95.4%, compared to 43.5% in control groups, with OR: 24.27 ((95% CI: 5.84, 100.82); *I*^2^ = 0%) (Fig. 6).

**Fig. 6.** Risk of TMD in Migraine—Sensitive diagnostic criteria. CI: confidence intervals.

##### 3.3.2.2 Overall TMD risk in tension-type headache—sensitive diagnostic criteria

8 studies analysed the prevalence of TMD diagnosis in TTH patients compared to non-headache subjects. Study characteristics varied regarding setting, data collection, diagnostic criteria and modalities. One study distinguished between Frequent episodic TTH (FETTH) and chronic TTH (CTTH) [34]. Due to the low frequency of CTTH prevalence, related data was not presented by the authors. One study [31] distinguished between TTH and probable TTH.

The pooled prevalence of TMD was 7.9% in TTH patients, whereas the prevalence of TMD in non-headache population was 0.3%, with OR 4.45 ((95% CI: 2.63, 7.53); *I*^2^ = 86%).

Two studies published by the same research group [29, 37], one population-based and one clinic-based, considering ETTH diagnoses based on questionnaires, reported pooled prevalence of TMD of 40.7% in headache patients, and 17.8% in non-headache subjects (OR 3.06 (95% CI: 1.90, 4.94); *I*^2^ = 21%).

One study regarding CTTH, based on a community database of twins [40], reported TMD prevalence of 12.4% in headache patients and 4.4% in non-headache subjects (OR 3.10 (95% CI: 2.14, 4.49)) (Fig. 7).

**Fig. 7.** Risk of TMD in TTH—Sensitive diagnostic criteria. CI: confidence intervals.

#### 3.3.3 Risk of specific TMD diagnoses in headaches

##### 3.3.3.1 Arthrogenous TMD in headache patients

4 studies [28, 29, 33, 34] assessed the prevalence of Arthrogenous TMD in migraineurs, which was reported as 3.1%, compared to 1.8% in control groups, without statistical differences between groups. Arthrogenous TMD was present in 8.8% of TTH subjects evaluated in 3 studies [28, 29, 34], while the control group’s prevalence was 8.7%. No difference was observed.

Headache patients did not show to be at higher risk of Arthrogenous TMD (OR 0.96 (95% CI: 0.54, 1.71); *I*^2^ = 33%) compared to non-headache samples.

##### 3.3.3.2 Myogenous TMD in headache patients

5 studies [28, 29, 30, 33, 34] compared the prevalence of myogenous TMD in migraine, which was observed in 19.1% of the study group, compared to 10.7% in control groups (OR 1.96 (95% CI: 1.56, 2.45)). Myogenous TMD was also more prevalent in 3 [28, 29, 34] studies including TTH subjects (12.2%) compared to control groups (4.3%) (OR 3.03 (95% CI: 1.29, 7.15)).

Pooled risk of myogenous TMD in primary headache subjects was calculated as OR 1.97 ((95% CI: 1.57, 2.46) with *I*^2^ = 0%. (Pooled prevalence 18.03% *vs.* 10.71%).

##### 3.3.3.3 Combined joint and myogenous TMD in headache patients

5 studies [28, 29, 30, 33, 34] assessed the prevalence of combined TMD in migraineurs, which was calculated as 19.5%, compared to 2.5% in control groups (OR 2.74 (1.40, 5.36); *I*^2^ = 63%). Combined TMD was also present in 40.1% of TTH subjects evaluated in 3 studies [28, 29, 34], while control groups prevalence was 21.2% (OR 2.81 (95% CI: 1.77, 4.46); *I*^2^ = 47%).

##### 3.3.3.4 Painful TMD in headache patients

Pooled results from 8 studies [28, 30, 31, 32, 33, 34, 37, 38] resulted in 16.8% prevalence of painful TMD in headache subjects, compared to 3.0% in non-headache populations (OR 5.31 (95% CI: 2.96, 9.54); *I*^2^ = 96%).

Prevalence of Painful TMD was 16.3% in migraineur subjects, compared to 3.0% in non-headache populations (7 studies; OR 4.66 (95% CI: 2.49, 8.70); *I*^2^ = 96%).

5 studies showed a prevalence of 43.0% of painful TMD in TTH patients, compared to 15.1% in control groups (OR 4.94 (95% CI: 2.02, 12.06); *I*^2^ = 86%).

##### 3.3.3.5 Non-Painful TMD in headache patients

Data from 2 studies [31, 32] showed a pooled 31.3% prevalence of non-painful TMD in headache patients, compared to 36.3% in non-headache subjects (OR 1.10 (95% CI: 0.28, 4.26); *I*^2^ = 78%) (Fig. 8).

**Fig. 8.** Risk of painful and non-painful TMD in Headache patients—Sensitive diagnostic criteria. CI: confidence intervals.

## 4. Discussion

Overall prevalence of TMD is higher in subjects with migraine and/or TTH than in non-headache individuals. Specifically, based on a subgroup analysis of studies based on reference diagnostic procedures, painful TMDs were more prevalent in TTH (OR 13.36 (95% CI: 3.01, 59.29)) and migraine (OR 6.81 (95% CI: 3.50, 13.24)) subjects, whereas arthrogenous disorders were not.

These findings support a clinical relationship between TMDs and primary headaches. Studies have also shown a higher prevalence of subclinical signs and symptoms, such as masticatory muscle and TMJ tenderness, in primary headache patients [41, 42, 43]. Moreover, the presence and higher intensity of temporomandibular pain in headache patients are associated with higher headache intensity, frequency, disability and medication intake [28, 33, 36]. In our review we observed the highest prevalence in chronic migraine patients (OR 24.27). Therefore, these findings may have a high clinical impact on the assessment and management of patients with headaches.

Migraine, TTH and TMDs, as different entities, share several mechanisms in pain processing, and central sensitization appears to play a major role in this process. The trigeminocervical complex mediates both head and face pain, therefore regional sensitization due to peripheral or central factors can facilitate, aggravate, or perpetuate craniofacial pain [44]. Allodynia or reduced pressure pain threshold (PPT) have been observed on trigeminal and C1–C3 segments in both headaches and TMD populations, with higher values in patients with both these conditions [45], supporting TMD as perpetuating or aggravating factors of headaches.

History of micro-trauma (bruxism) or macro-trauma (facial trauma) in craniofacial anatomical regions are also reported in both these populations, suggesting a role of peripheral inputs in painful symptoms [46]. From a clinical point of view, a higher prevalence of neck pain and disability or otologic symptoms [39, 47], suggests a segmental involvement and clinical overlap in describing these clinical presentations.

However, pain processing involving supraspinal mechanisms also appears to play a role in these relationships. High prevalence of widespread and systemic painful comorbidity and structural and functional changes on MRI have been observed in both these populations [38, 48, 49, 50, 51].

Migraine and TTH are primary headaches in nature, therefore capable of amplifying peripheral nociception.

Female subjects have shown a higher prevalence of TTH, migraine and/or TMDs, compared to males [16, 52]. Such prevalence has been previously attributed to several factors, including hormonal factors (*e.g.*, estrogens), due to the higher TMD prevalence and dysfunction in reproductive age [53]. However, although the link between hormones and headaches is well established, the literature lacks evidence regarding hormonal roles in TMDs.

Headache populations have been reported to suffer from depressive symptoms, anxiety and emotional distress [54]. Psychological aspects have been observed as key factors in subjects with TMD, to the extent, the Research Diagnostic Criteria for Temporomandibular Disorders (RDC/TMD) [55], and later on the DC/TMD comprise the Axis II, a specific assessment of psychological status and pain-related disability [15]. They have been linked to craniofacial pain in a bidirectional relationship. Painful symptoms can be easily considered as responsible for the psychological dimensions of pain experience. On the other hand, psychosocial impairments have been linked to higher perceived distress and disability in cross-sectional studies [56] and represent a risk factor for first-onset TMDs [57] as well as for poor prognosis [58].

Higher psychological distress has also been linked to oral habits (*e.g.*, onychophagia, chewing-gum use) and awake bruxism [59]. Such behaviours, observed in both headache and TMD patients [32, 60], can be enhanced by higher psychological distress [61, 62, 63], with or without painful symptoms. They can also represent a peripheral cause of nociception or a perpetuating factor due to masticatory muscles and TMJ overload [64, 65].

Most of these factors, as well as other aspects related to respiratory or oral health (*e.g.*, obstructive sleep apnea), could play as confounders of the causative association between headaches and TMDs. However, such features were not controlled in most of the included studies, and the methodological quality of the available literature regarding these topics is low, therefore no firm conclusion can be drawn [31, 32, 66, 67, 68].

Painful TMD may contribute to primary headache clinical presentation [69]. Even though studies on the efficacy of TMD management (*e.g.*, oral splint therapy) in primary headache patients have shown unsatisfying results [70], TMD treatment could be useful in patients with this specific comorbidity as an approach to clean the clinical picture. Preliminary findings appear promising on this topic [71]. On the other hand, a program of 10 weeks of Propranolol (Beta-blockage) has shown to be effective in improving TMD pain in subjects with both TMD and migraine [72].

Arthrogenous TMD and non-painful TMD did not appear to be associated with primary headaches. As described in the DC/TMD classification system, joint disorders include anatomical and biomechanical changes in the normal anatomy of the temporomandibular joint, mainly regarding disc displacements or degenerative changes. This can be diagnosed through clinical examination, mainly through the identification of joint sounds and functional limitations. The gold standard in the diagnosis of joint TMD is medical imaging (Magnetic resonance imaging for soft tissue, computed tomography for bone changes) [15] (Schiffman 2014), which should be prescribed upon need [73, 74]. This category does not mandatorily imply the presence of painful symptoms reported by the patient or reproduced by the examiner. Such findings support the relatively scarce role of structural alteration of temporomandibular joints in most orofacial pain presentations, both in primary headaches and in pain of temporomandibular origin.

The different clinical impact of painful and structural TMD has been an important focus in implementing high-quality management of patients with orofacial pain, in a path that led to the development of the International Classification of Orofacial Pain [20, 75, 76]. The process that led to the publication of ICOP was much needed due to the high heterogeneity observed in studies on the face and head pain, as we also could see developing this systematic review. From all studies included, only five reported the reference classification for both TMD (RDC/TMD, DC/TMD or ICOP) and headaches (ICHD-2 or ICHD-3) (Section 2 of the results). High heterogeneity in inclusion criteria however showed similar results between data from more sensitive or specific inclusion criteria, underlining the higher prevalence of painful TMD in migraine and TTH patients, but not for arthrogenous TMDs. These findings support our choice to include all studies referring to primary headaches and TMDs, intending to analyse and summarize the whole literature on this topic.

Studies regarding face or head pain without a specific diagnosis were excluded from this review. Headache as a symptom has been described as one of the most prevalent comorbidities in patients with TMD. Moreover, even if isolated face pain is rare in primary headaches [77, 78], face signs and symptoms, such as fatigue, reduced pressure pain threshold, discomfort or perceived difficulties in the mandibular movement are often reported by headache patients.

We excluded IETTH from our review. Due to the high prevalence and limited impact on the quality of life and disability of these conditions our analysis could have been impacted, reducing the external validity of our findings. We also excluded data regarding Chronic Daily Headaches. This diagnostic term is not included in the ICHD-3. Since this term could encompass several headache disorders (*e.g.*, chronic or transformed migraine, chronic tension-type headache (CTTH), new daily persistent headache and hemicrania continua) [79], we considered such studies not suitable for our review.

Preliminary results from this SR were presented as posters at the International Headache Society and European Headache Federation Joint Congress 2021 and the American Headache Society Congress 2021.

## 5. Limitations

This systematic review presents exclusively cross-sectional studies, which design does not imply causation between these conditions. However, recent studies have shown that suffering from migraine, mixed headache or headache frequency at baseline increased the risk of developing TMD at 5 years follow-up [80], and a diagnosis of TMD has been described as a risk factor for the first diagnosis of migraine in 5 years follow up [81].

Data from studies included could be biased in several ways, due to heterogeneity in setting, diagnostic procedure and data collection heterogeneity. Our choice for a subgroup analysis based on diagnostic procedure, and the presence of painful/non-painful TMD however appeared to decrease the heterogeneity in the results of our meta-analysis.

## 6. Conclusions

TTH and migraine patients appear to be more prone to temporomandibular disorders compared to non-headache subjects. Possible comorbidities should be considered in the assessment of patients with craniofacial pain.

## 7. Highlights

• Patients with migraine or Tension-type headaches have a higher risk of painful, myogenous or mixed temporomandibular disorders compared to non-headache subjects.

• Prevalence of arthrogenous TMD or non-painful TMD was similar between headache and non-headache populations.

• These findings should be considered during assessment and management of patients with migraine or tension-type headaches.

## Supplementary Material


