# Supplementary material for: Temporomandibular disorders in migraine and tension-type headache patients: a systematic review with meta-analysis
Source: J Oral Facial Pain Headache. 2024 Jun 12;38(2):11–24. doi: 10.22514/jofph.2024.011 (PMC11810655; doi:10.22514/jofph.2024.011)
Supplement: Supplementary file 1 [file Supplementary-material.docx]

Supplementary material

EMBASE

(‘migraine’/exp OR migraine OR ‘tension headache’/exp OR ‘tension headache’ OR ‘tension-type headache’/exp OR ‘tension-type headache’) AND (temporomandibular OR craniomandibular OR craniofacial OR orofacial)

MEDLINE

(migraine or “tension headache” or “tension-type headache”) and (temporomandibular or craniomandibular or craniofacial or orofacial)

CENTRAL

(migraine or “tension headache” or “tension-type headache”) and (temporomandibular or craniomandibular or craniofacial or orofacial)
